# Unique immune and inflammatory cytokine profiles may define long COVID syndrome

**DOI:** 10.1007/s10238-023-01065-6

**Published:** 2023-04-16

**Authors:** Lao-Tzu Allan-Blitz, Omid Akbari, Noah Kojima, Edwyn Saavedra, Prithivi Chellamuthu, Nicholas Denny, Melanie A. MacMullan, Victoria Hess, Maria Shacreaw, Matthew Brobeck, Frederick Turner, Vladimir I. Slepnev, Albina Ibrayeva, Jeffrey D. Klausner

**Affiliations:** 1 Division of Global Health Equity: Department of Medicine, Brigham and Women’s Hospital, 75 Francis Street, Boston, MA 02115 USA; 2 Department of Population and Public Health Sciences, Keck School of Medicine, University of Southern California, Los Angeles, CA 90033 USA; 3 Department of Medicine, University of California Los Angeles, Los Angeles, CA USA; 4 Curative Inc., San Dimas, CA USA

**Keywords:** SARS-CoV-2, Long COVID, Biomarkers, Inflammatory cytokines, Antibodies

## Abstract

**Purpose:**

Long COVID is estimated to occur in 5–10% of individuals after acute SARS-CoV-2 infection. However, the pathophysiology driving the disease process is poorly understood.

**Methods:**

We evaluated urine and plasma inflammatory and immune cytokine profiles in 33 individuals with long COVID compared to 33 who were asymptomatic and recovered, and 34 without prior infection.

**Results:**

Mean urinary leukotriene E4 was significantly elevated among individuals with long COVID compared to asymptomatic and recovered individuals (mean difference 774.2 pg/mL; SD 335.7) and individuals without prior SARS-CoV-2 infection (mean difference 503.1 pg/ml; SD 467.7). Plasma chemokine ligand 6 levels were elevated among individuals with long COVID compared to individuals with no prior SARS-CoV-2 infection (mean difference 0.59 units; SD 0.42). We found no significant difference in angiotensin-converting enzyme 2 antibody levels. Plasma tumor necrosis factor receptor-associated factor 2 (TRAF2) levels were reduced among individuals with long COVID compared to individuals who were asymptomatic and recovered (mean difference = 0.6 units, SD 0.46). Similarly, the mean level of Sarcoma Homology 2-B adapter protein 3 was 3.3 units (SD 1.24) among individuals with long COVID, lower than 4.2 units (SD 1.1) among individuals with recovered, asymptomatic COVID.

**Conclusion:**

Our findings suggest that further studies should be conducted to evaluate the role of leukotriene E4 as a potential biomarker for a diagnostic test. Furthermore, based on reductions in TRAF2, long COVID may be driven in part by impaired TRAF2-dependent immune-mediated inflammation and potentially immune exhaustion.

## Introduction

After initial SARS-CoV-2 infection, an estimated 5–10% of individuals develop persistent symptoms [1]. The World Health Organization defines “long COVID” as symptoms lasting for at least 2 months, at least 3 months after the onset of COVID-19 disease or a positive SARS-CoV-2 test, without evidence of an alternative diagnosis [2]. Clinical manifestations of long COVID include fatigue, shortness of breath, and cognitive changes, among many other symptoms [2]. The underlying pathophysiologic mechanisms, however, of long COVID and other post-infectious syndromes are poorly understood, and no objective diagnostic tests exist.

Some postulate that long COVID results from  autoimmune disease due to the development of angiotensin-converting enzyme 2 (ACE-2) autoantibodies which reduce angiotensin 2 activity and increase angiotensin II, a pro-inflammatory molecule [3]. Additionally, others have observed sustained fluctuations in non-specific peripheral inflammatory and immune-associated cytokines as well as elevations in neuronal-enriched extracellular vesical proteins among individuals with long COVID [4, 5], suggesting persistent low-grade neuroinflammation [6]. More recent work has highlighted differences in innate immune cell functioning and elevated expression of peripheral pro-inflammatory cytokines among individuals with long COVID [7]. In contrast, findings of reduced markers of T-cell-mediated immunity have led some experts to hypothesize an “immune exhaustion,” preventing healing of tissues injured during acute infection [8–10].

While identifying a panel of biomarkers has been proposed as a means of diagnosing long COVID [7], no specific biomarkers have been identified that consistently distinguish patients with long COVID from those without. Such biomarkers would be fundamental to our understanding of the pathophysiology behind long COVID as well as provide a direction toward laboratory diagnostic testing. To explore unique biomarkers in patients with long COVID, we evaluated a panel of inflammatory and immune-mediated cytokines among individuals with long COVID compared to recovered individuals without long COVID symptoms and individuals without prior SARS-CoV-2 infection.

## Methods

From November 18, 2021 to May 7, 2022, we contacted individuals ≥ 18 years of age residing in the greater Los Angeles area who had been tested by Curative Inc., a large national testing laboratory. We enrolled individuals with a positive polymerase chain reaction (PCR) test for SARS-CoV-2 at least 4 months previously as well as individuals without knowledge of a prior SARS-CoV-2 infection. Participants gave informed consent to participate in the study and were surveyed on sociodemographic factors, participant-reported pre-existing conditions, and persistent symptoms.

Long COVID was defined in individuals with a positive PCR at least 6 months prior to study enrollment and two or more of the following persistent symptoms: loss of taste or smell, mental fogginess, headache or blurry vision, fatigue, sleep disturbances, dizziness, irregularities in heartbeat, shortness of breath, muscle aches, pain (abdominal, chest, or joints), cough, fevers, or gastrointestinal issues. Recovered asymptomatic COVID was defined in individuals with a positive SARS-CoV-2 PCR test at least 4 months previously without persistent symptoms. SARS-CoV-2-negative controls were defined in individuals without a prior history of infection or disease and a negative SARS-CoV-2 nucleocapsid (N)-protein IgG antibody test result (GSD™ SARS-CoV-2 IgG ELISA Test Kit; Gold Standard Diagnostics, Davis, CA, USA).

Urine and blood specimens were collected at study participants’ residence by a trained phlebotomist. Blood samples (15 mL) were transported on ice in Separation Transport Tubes (BD; Franklin Lakes, NJ), while urine samples (40 mL) were collected in a Falcon tube and transported on ice. Urine samples were processed within 2–3 h of collection, and all specimens had no more than one freeze thaw cycle to avoid analyte degradation.

We tested urine specimens for leukotriene E4 (Cayman Chemical, Ann Arbor, MI, USA) and N-methylhistamine (Eagle Biosciences, Amherst, MA, USA), aliquoting 50 µL of urine for the leukotriene E4 kit and 20 µL of urine for the N-methylhistamine kit in accordance with the package insert. We tested plasma samples for ACE-2 antibodies (CellTrend GmbH, Luckenwalde, Germany), tryptase (Lifespan Biosciences, Seattle, WA, USA), prostaglandin (Cusabio Technology LLC., Houston, TX, USA), SARS-CoV-2 spike (S)-protein IgG antibodies (Euroimmun Inc., Lübeck, Germany), SARS-CoV-2 nucleocapsid (N)-protein IgG antibodies, and total SARS-CoV-2 IgG antibodies (Gold Standard Diagnostics, Davis, CA, USA).

Enzyme immunoassays for each individual cytokine were used according to the manufacturer’s instructions and read on the Thunderbolt^®^ Analyzer (Gold Standard Diagnostics, Davis, CA, USA). Plasma specimens were also sent to Olink Proteomics Inc. (Boston, MA) for testing on their Olink^®^ Target 96 Inflammation and Immune Response panels, with results reported as units standardized to each individual assay.

We compared mean levels of each cytokine among individuals with long COVID to mean levels among asymptomatic recovered individuals and individuals with no prior SARS-CoV-2 infection, using Student’s t-tests for parametric data and Welch’s tests for nonparametric data. For the SARS-CoV-2 antibodies (S-protein, N-protein, and total IgG levels), we standardized those values by the number of days since infection to account for differences in the time to enrollment from the date of infection between those with long COVID and those recovered and asymptomatic. Given the exploratory nature of the study, we did not adjust for multiple comparisons. The study received institutional review board approval from the Advarra institutional review committee (IRB: Pro00053732; Advarra, Columbia, MD, USA).

## Results

Among 100 participants, 33 (33.0%) had long COVID, 33 (33.0%) were asymptomatic and recovered, and 34 (34.0%) had no current or prior COVID-19 disease, confirmed by the absence of SARS-CoV-2 nucleoprotein IgG antibody. The median time since infection among those with long COVID was 370 days (IQR 46.5), compared to 190 days (IQR 164.3) among those asymptomatic recovered. Of those with long COVID, 21 (63.6%) were women, while 15 (45.5%) were women among those recovered and asymptomatic, and 16 (47.1%) were women among those without prior SARS-CoV-2 infection. In each of the three groups, 100% of participants reported prior immunization against SARS-CoV-2. The most common symptoms reported among those with long COVID was fatigue (78.8%) followed by mental fogginess (69.7%). There were three individuals with prior autoimmune diseases (hypothyroidism, inflammatory bowel disease, and rheumatoid arthritis), two of whom were among the cohort with long COVID.

The Table 1 shows the comparison of measured cytokine levels for each of the three groups. Notably, from urine samples, leukotriene E4 was significantly elevated among individuals with long COVID compared to asymptomatic and recovered individuals and individuals without prior SARS-CoV-2 infection.

**Table 1:** Comparison of urine and plasma cytokine profiles among individuals with long COVID to asymptomatic, recovered individuals and individuals with no prior SARS-CoV-2 infection, Los Angeles community-based study sample, 2021–2022

Cytokine	No. of tested	Long COVID	Recovered COVID		No SARS-CoV-2 infection	
		Average concentration (SD)	Average concentration (SD)	Mean difference (SD) (LC vs. RC)	Average concentration (SD)	Mean difference (SD) (LC vs. NC)
*Urine samples*						
Leukotriene E4	96	2372.2 pg/mL (1259.8)	1598.0 pg/mL (1398.9)	**744.2 (335.7)**	1869.1 pg/mL (2362.3)	**503.1 (467.7)**
N-methyl-histamine	78	99.6 ng/mL (75.8)	36.3 ng/mL (36.5)	**63.3 (16.2)**	56.3 ng/mL (54.5)	43.3 (17.9)
*Plasma samples*						
ACE-2 Antibody	93	746.4 U/mL (1414.6)	542.0 U/mL (544.1)	204.4 (268.5)	389.8 U/mL (473.7)	356.6 (264.6)
Tryptase	86	0.5 ng/ml (0.3)	0.7 ng/mL (0.4)	− 0.2 (0.1)	0.4 ng/mL (0.3)	0.1 (0.1)
Prostaglandin	77	210.1 pg/L (246.5)	263.9 pg/mL (135.7)	− **53.8 (56.3)**	253.6 pg/mL (313.6)	− 43.5 (77.9)
S-protein Antibody^†^	98	9633.1 ng/mL (10,722.4)	24,863.0 ng/mL (29,406.1)	− 15,229.9 (5601.6)	25,756.6 ng/mL (38,642.8)	− 15,229.9 (6885.0)
N-protein Antibody^†^	96	726.4 ng/mL (1337.9)	2990.5 ng/mL (6104.1)	− **2264.1 (1121.6)**	0 ng/mL (0)	**726.4 (236.5)**
Total IgG^†^	79	3,154,453.6 ng/mL (4,486,835.4)	818,253.9 ng/mL (693,861.4)	2,336,199.7 (858,781.7)	1,420,638.0 ng/mL (1,452,631.2)	1,733,815.6 (916,097.0)

Long COVID (LC); recovered COVID (RC); and no SARS-CoV-2 infection (NC). Bold cells indicate significant differences at the 0.05 level

^†^Corrected for days since infection

Plasma analysis showed no difference in  mean concentration of ACE-2 antibodies among individuals with long COVID, asymptomatic and recovered individuals, or individuals without prior infection. SARS-CoV-2 N-protein IgG levels among individuals with long COVID were lower compared to those asymptomatic and recovered (mean difference 2264.1 ng/mL; SD 1121.6), standardized by days since infection.

**Fig. 1:**
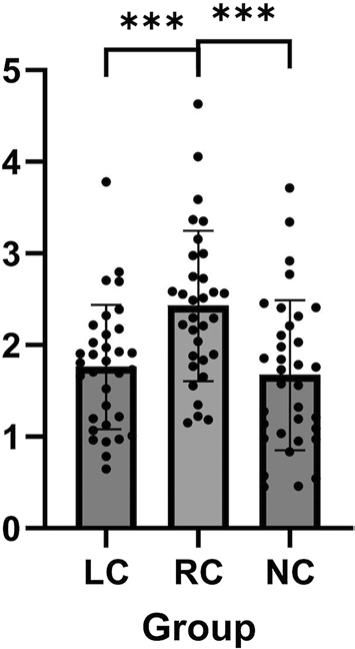
Mean levels of serum tumor necrosis factor receptor-associated factor 2 from a panel of immune cytokines among individuals with long COVID, recovered COVID, and no prior SARS-CoV-2 infection. The figure shows the mean differences and 95% confidence intervals between serum tumor necrosis factor receptor-associated factor 2 levels among individuals with long COVID (LC), recovered COVID (RC), and no prior SARS-CoV-2 infection (NC). ***Difference in means was statistically significant

Upon analysis of the Olink immune response panel, tumor necrosis factor receptor-associated factor 2 (TRAF2) showed a mean level of 1.8 standardized units (SD 0.69) among individuals with long COVID (*n* = 33), which was significantly reduced compared to a mean level of 2.4 units (SD 0.81) among individuals who were asymptomatic and recovered from COVID (*n* = 32), (mean difference = 0.6 units, SD 0.46). Figure 1 shows the distribution and the mean levels of TRAF2 among the three groups. Similarly, the mean level of Sarcoma Homology 2-B adapter protein 3 (SH2B3) was 3.3 units (SD 1.24) among individuals with long COVID, which was lower compared to 4.2 units (SD 1.1) among individuals with recovered, asymptomatic COVID. There were no significant differences in the average levels of TRAF2 or SH2B3 among individuals with long COVID compared to individuals without prior SARS-CoV-2 infection.

Findings from the Olink inflammatory response panel also demonstrated elevation in chemokine ligand 6 (CXCL6) among individuals with long COVID compared to individuals with no prior SARS-CoV-2 infection (mean difference 0.59 units; SD 0.42), as well as between asymptomatic and recovered individuals compared to individuals without prior SARS-CoV-2 infection (mean difference 0.62 units; SD 0.44); however, there was no significant difference in mean levels of CXCL6 among individuals with long COVID compared to asymptomatic and recovered individuals.

## Discussion

We explored variations in an array of inflammatory and immunomodulatory measures among individuals with long COVID compared to asymptomatic and recovered individuals and individuals with no prior SARS-CoV-2 infection. We found notable elevations in urinary leukotriene E4 and plasma CXCL6 levels among individuals with long COVID. Plasma TRAF2 and SH2B3 levels were also notably reduced among individuals with long COVID compared to individuals asymptomatic and recovered from COVID. There was no significant difference in levels of ACE-2 autoantibodies among the three groups.

Leukotriene E4 has been shown to induce vascular permeability and potentiate mucosal inflammation through mast cell recruitment and activation [11]. Leukotriene E4 is specifically associated with mediating inflammation in the pulmonary tissue [12]. The elevation of urinary leukotriene E4 among individuals with long COVID may serve as a marker of low-level ongoing pulmonary inflammation. Similarly, the elevation of CXCL6 in the plasma of participants with long COVID may reflect non-specific inflammation from tissue injury, as CXCL6 functions as a pro-inflammatory cytokine attracting neutrophils [13] as well as in regulating cell proliferation and apoptosis after ischemic injuries [14]. Persistence of SARS-CoV-2 infection among patients with long COVID has been suggested by numerous reports finding viral proteins or RNA in multiple organs, including the lungs [15–20]. Significant differences in urinary Leukotriene E4  levels might provide the basis for a diagnostic assay. Further studies in larger populations would be necessary to validate its use as a marker and demonstrate its performance at distinguishing those with and without long COVID.

Further, there was no significant difference in levels of ACE-2 antibody among individuals with long COVID compared to the other groups. That finding contrasts a prior study reporting elevated ACE-2 autoantibodies among 81% (26/32) of patients with long COVID compared to 5% (1/20) among outpatient participants with prior SARS-CoV-2 infection who were asymptomatic and recovered [3]. Our findings suggest that ACE-2 antibody levels are insufficient to distinguish long COVID from asymptomatic and recovered individuals or from individuals without prior SARS-CoV-2 infection. Notably, the severity of initial symptoms may be correlated with ACE-2 antibody levels [3]; however, we did not have access to data on symptoms from the initial infection.

TRAF2 is a mediator of intracellular signaling downstream of the TNF-α superfamily, important in mediating cell survival and normal adaptive immune responses [21]. Recent work has demonstrated that the loss of T-cell TRAF2 function results in constitutive activation of an inflammatory pathway, which may drive fatal inflammation in mice, independently of TNF-α signaling [21]. Further, inhibition of TRAF2 may result in impaired peripheral regulatory T-cell functioning [22], which may contribute to the induction of autoimmunity and abrogation of peripheral immune tolerance. Autoimmunity has also been invoked as a potential contributor to long COVID symptoms [9]. The notable attenuation in TRAF2 activity among individuals with long COVID may also indicate an overall reduced type 1-mediated immune response, consistent with recent work noting reduced IL6 and other cytokines suggesting an immune exhaustion among individuals with long COVID [8, 9].

**Fig. 2:**
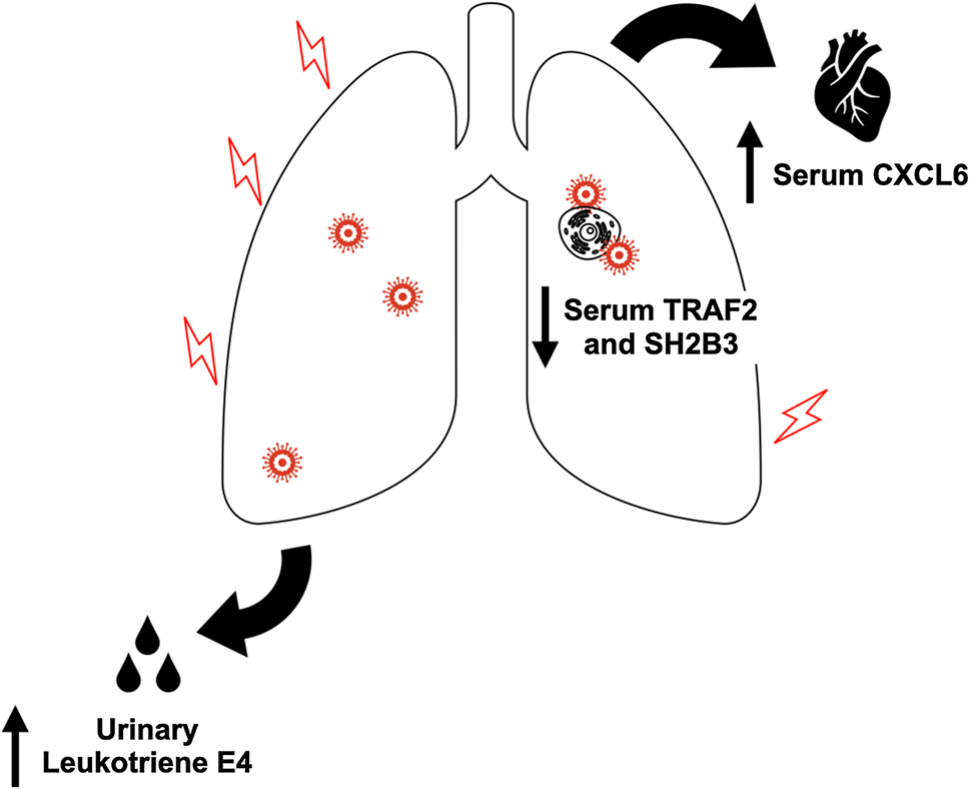
Cytokine derangements attributed to long COVID. The figure shows four different cytokine derangements identified among patients with long COVID. First, leukotriene E4, a marker of tissue inflammation, is elevated and detected in the urine. Second, CXCL6, another marker of pulmonary injury, is elevated in the serum. Finally, T-cell production of TRAF2 and SH2B3 is reduced systemically

Reductions in SH2B3 have been shown to potentiate expansion of early hematopoietic progenitor cells and increased lymphocyte production [23]; thus, the observed reduction in SH2B3 in the plasma of patients with long COVID in our study may be a non-specific marker of reduced underlying inflammation, again consistent with immune exhaustion. One unifying mechanistic pathway explaining our findings is depicted in Fig. 2. Tissue injury, likely pulmonary, results in elevated urinary leukotriene E4 as well as serum CXCL6 consequent to tissue inflammation. Global immune function attenuation, consistent with the immune exhaustion hypothesis, results in reduced TRAF2 and SH2B3. Further evaluation of the potential pathophysiologic mechanism of inflammation underlying long COVID should evaluate the possible roles of the above cytokines, as well as others previously reported [5, 8–10].

Importantly, as this was an exploratory analysis, we did not adjust for multiple comparisons, increasing the probability of finding significant differences observed by chance alone. Therefore, our results are hypothesis generating. Additionally, our sample size was small and from a single geographical region. Similarly, the low prevalence of concurrent autoimmune conditions in our cohort precluded evaluating that as a possible explanation for the differences in cytokine profiles observed, which should be investigated further. Testing among larger and more diverse populations is warranted. Finally, the median time since infection in those with long COVID among our population was longer than in those asymptomatic and recovered, likely due to the study design; thus, it remains possible the differences observed may change over time, supporting the need for replication of our findings. However, our findings provide valuable groundwork and hypotheses for future research into the pathophysiology of and diagnostic test development for long COVID. We feel that the above limitations do not negate the importance of our findings.

## Conclusion

Among individuals with long COVID, urinary leukotriene E4 as well as plasma CXCL6 were persistently elevated compared to both individuals asymptomatic and recovered and those with no prior evidence of infection, while TRAF2 and SH2B3 were reduced compared to individuals who were asymptomatic and recovered. Notably, we found no significant difference in mean concentration of ACE-2 antibodies among individuals with long COVID and the other groups. Our findings may provide insights into the pathophysiology behind long COVID, as well as inform efforts to develop an objective diagnostic test. Further research is warranted.

## Data Availability

Data are available upon request.
